# Therapeutic

**Published:** 1867-08

**Authors:** 


### THERAPEUTIC.

We observed not long since some most beautiful therapeutic preparations for the Dentist's use, prepared and put up by S. S. White. Among them are the "Styptic Colloid" "Carbolic Acid and Glycerin" and "Iodine and Glycerin." These are preparations in constant use by the Dentist; and he has them here prepared in the very best manner, and he obtains them with no further trouble than the purchase. It is gratifying to know, that whatever advances our profession may make, there is at least one man, who almost anticipates any requirement of such advances, and immediately furnishes the necessary facilities.

The preparations are, we presume, to be had at the Dental Depots.

